# Effect of diacerein as an add-on to metformin in patients with type 2 diabetes mellitus and inadequate glycemic control

**DOI:** 10.1590/2359-3997000000242

**Published:** 2017-01-27

**Authors:** Miriam Méndez-del Villar, Esperanza Martínez-Abundis, Rafael O. Preciado-Márquez, Manuel González-Ortiz

**Affiliations:** 1 Institute of Experimental and Clinical Therapeutics Health Science University Center University of Guadalajara Guadalajara Mexico Institute of Experimental and Clinical Therapeutics, Physiology Department, Health Science University Center, University of Guadalajara, Guadalajara, Mexico

**Keywords:** Diacerein, metformin, glycemic control, type 2 diabetes mellitus

## Abstract

**Objective:**

To evaluate the effect of diacerein as an add-on to metformin in patients with type 2 diabetes mellitus (T2DM) and inadequate glycemic control.

**Materials and methods:**

A randomized, double-blind, placebo-controlled clinical trial was carried out on 12 patients with T2DM and inadequate glycemic control [glycated hemoglobin A1c (A1C) ≥ 7%] with metformin as monotherapy (≥ 1500 mg per day) for at least the previous 90 days. Fasting and postprandial glucose were measured before and after the pharmacological intervention. A1C, lipid profile, creatinine and uric acid were also evaluated. After randomization, all patients continued with their dose of metformin. Six subjects received placebo and the other six volunteers took diacerein. Data were tested using the Wilcoxon signed-rank, Mann-Whitney U and chi-square tests. The Institutional Ethics Committee approved the study protocol.

**Results:**

After 90 days of diacerein as an add-on to metformin, there was a significant decrease in fasting glucose (196 ± 79 vs. 149 ± 70 mg/dL, p < 0.05), postprandial glucose (262 ± 99 vs. 187 ± 70 mg/dlL, p < 0.05) and A1C (8.4 ± 2.0 vs. 6.7 ± 1.7 %, p < 0.05).

**Conclusions:**

Diacerein as an add-on to metformin in patients with T2DM improved their glycemic control.

## INTRODUCTION

Adipose tissue is considered an endocrine organ that secretes various adipokines involved in metabolic regulation and inflammatory processes (1). Dysregulation of endocrine function and inflammation of adipose tissue induce a systemic inflammation and insulin resistance in patients with overweight and obesity, which may lead to the development of type 2 diabetes mellitus (T2DM) (2). Tumor necrosis factor-α (TNF-α) and interleukin (IL)-1β have been involved in apoptosis of pancreatic β-cells, decreasing insulin secretion with the consequent hyperglycemia characteristic of T2DM (3,4).

Diacerein is an anthraquinone derivative whose active metabolite is rhein. It is considered a symptomatic slow-acting drug for the treatment of certain articular diseases such as osteoarthritis with anti-inflammatory, anti-catabolic and pro-anabolic properties on cartilage and synovial membrane. It has also recently been shown to have protective effects against subchondral bone remodeling. The use of diacerein is associated with common gastrointestinal disorders such as soft stools and diarrhea, common mild skin reactions, and, uncommonly, hepatobiliary disorders. No serious adverse events have been associated with its long-term use (5).

Metformin is an oral glucose-lowering agent of the biguanide family and is derived from *Galega officinalis*. It currently is the first-choice drug for T2DM treatment because of its effectiveness, low cost and safety (6). Its antihyperglycemic properties are due to its effects on various tissues such as intestine, liver and skeletal muscle.

**Figure 1:**
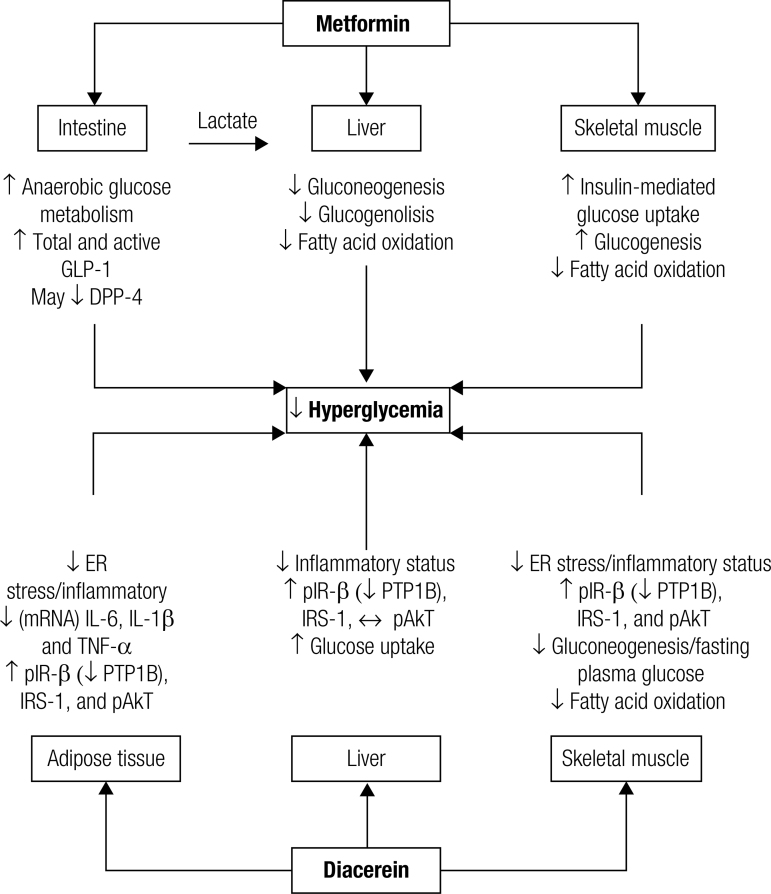
Mechanism of action of metformin and diacerein for glucose improvement (Ref. 6-9).

GLP-1: glucagon like peptide 1; DPP-4: dipeptidyl-peptidase 4; ER: endoplasmic reticulum; mRNA: messenger RNA; IL-6: interleukin 6; IL-1β: interleukin 1β; TNF-α: tumor necrosis factor α; pIR-β: phosphorylated insulin receptor type β; PTP1B: protein-tyrosine phosphatase 1B; IRS-1: insulin receptor substrate 1; pAkt: activated protein kinase B.

Meanwhile, the anti-inflammatory properties of diacerein due to the decrease of some cytokine concentrations, mainly TNF-α and IL-1β, may be responsible for insulin resistance improvement (7) (Figure 1) (6-9).

Due to the anti-inflammatory effects of diacerein, it could be considered as a pharmacological option to counter the systemic inflammation and insulin resistance characteristic of patients with obesity (8). Administration in drug-naïve patients with T2DM significantly increases the first, late, and total insulin secretion phases, improving metabolic control (10).

Several pharmacological options are available for the treatment of T2DM with different mechanisms of action (11). In many cases the combination of therapies, which improve both insulin sensitivity and secretion, is recommended (11,12).

If diacerein improves insulin secretion in T2DM patients, it may be used in combination with an insulin sensitizer such as metformin, which is considered the first drug of choice for T2DM treatment. Therefore, the aim of this study was to evaluate the effect of diacerein as an add-on to metformin in patients with T2DM and inadequate glycemic control.

## MATERIALS AND METHODS

A randomized, double-blind, placebo-controlled clinical trial was performed including 12 patients with T2DM and inadequate glycemic control [glycated hemoglobin A1c (A1C) levels ≥ 7%] with metformin as monotherapy (≥ 1500 mg per day) for at least the previous 90 days. Subjects were between 30 and 60 years of age and with overweight or obesity according to body mass index (BMI) (25.0–34.9 kg/m^2^). Subjects were selected from the same residential area and socioeconomic status. No participant was excessively sedentary or engaged in strenuous physical activity. All individuals were nonsmokers and their body weight remained stable for at least 90 days prior to the study. Patients had no history of hepatic, renal, thyroid, or heart disease. Subjects denied use of any other medications that affect glucose metabolism during the previous 6 months. Patients were excluded if they were pregnant, breastfeeding, or had a known allergy to diacerein.

Patients were evaluated before and after the 90-day study period. All patients received general recommendations about their medical nutritional therapy and were instructed to not modify their usual physical activity. Tests were performed at 8:00 a.m. after a 10- to 12-h overnight fast.

Height and body weight were measured with individuals wearing light clothing and no shoes. Height was measured with subjects standing and head in Frankfort plane (when orbitale is at the same horizontal plane with tragion). Measurements were rounded to the nearest centimeter. Body weight was evaluated using a bioimpedance digital scale and results were reported in kilograms using a decimal. Fat mass, in kilograms, was evaluated through bioimpedance. BMI was calculated by dividing body weight (kg) by height squared (m^2^). Waist circumference was measured with a flexible steel certified Lufkin^®^ tape at the midpoint between the lowest rib and the iliac crest and was expressed in centimeters using a decimal. Blood pressure was measured three times at the left arm with a digital sphygmomanometer (Omron Hem-907 XL^®^) with the subject seated in a chair after a 5-min rest. The mean of the three measurements was considered as the value of systolic blood pressure and diastolic blood pressure expressed in mmHg.

Blood samples were taken under fasting conditions to measure serum fasting glucose (FG), total cholesterol (TC), triglycerides (TG), high-density lipoprotein cholesterol (HDL-C), creatinine and uric acid levels. Whole blood was collected for A1C concentrations. After the fasting blood sample, patients were requested to eat their breakfast as usual. A second blood sample 2 h after breakfast intake was taken to evaluate postprandial glucose (PPG) concentrations.

FG, TG, TC, creatinine and uric acid concentrations were measured by enzymatic colorimetric methods in an automated analyzer (Erba XL-100^®^). All determinations had intra- and inter-assay coefficients of variation < 1% and 2%, respectively. Whole blood A1C concentrations were determined using ion-exchange high-performance liquid chromatography technique (Bio-Rad Laboratories, Hercules, CA) with intra- and interassay coefficient of variation of 0.4 and 1.6%, respectively. Low-density lipoprotein cholesterol (LDL-C) levels were calculated using the Friedewald formula as follows: LDL-C = TC – HDL-C – (TG/5).

### Pharmacological administration

All patients received daily metformin (≥ 1500 mg per day) prior to the beginning of the study. Six patients were randomly assigned to receive 50 mg of diacerein (Representaciones e Investigaciones Médicas, S.A. de C.V., México City, Mexico) before breakfast for 15 days and for the remaining 75 days dose was titrated to receive 50 mg of diacerein before breakfast and dinner. The remaining six patients received placebo according to the same schedule. Simple random allocation was performed using a random number list.

### Statistical analyses

Sample size was calculated using a formula for clinical trials (13) with a statistical confidence of 95%, statistical power of 80%, standard deviation (SD) for FG of 27 mg/dL, and an expected between-group difference of at least 39 mg/dL for FG, obtaining a total of six patients for each group. For A1C and PPG, sample size calculation was lower. Values are presented as mean ± standard deviation. Data were tested using the Wilcoxon signed-rank test for intragroup differences. Mann-Whitney U test was used for intergroup differences and chi-square test was performed for qualitative determinations; p ≤ 0.05 was considered significant.

### Ethical considerations

The study protocol was reviewed and approved by the Institutional Ethics Committee and written informed consent was obtained from all volunteers.

## RESULTS

All patients who were eligible after enrollment completed the 90 days of the pharmacological intervention. Of the six patients from the metformin plus placebo group, five patients (83.3%) were female and one patient was male (16.7%). Mean age was 54.0 ± 3.5 years. In the metformin plus diacerein group, four patients (66.7%) were male and two patients were female (33.3%). Mean age was 41.3 ± 9.7 years. There were no significant differences between gender distribution between groups (p = 0.071) and between ages (p = 0.143). There were no significant differences between groups at baseline according to clinical and laboratory characteristics (Table 1).

**Table 1:** Characteristics before and after the interventions

	Metformin		Metformin plus diacerein	
	
	Baseline	90 days	Baseline	90 days
BMI (kg/m^2^)	32.0 ± 2.4	31.7 ± 2.0	32.5 ± 4.2	32.1 ± 3.7
Fat mass (kg)	31.7 ± 6.6	31.2 ± 5.0	30.7 ± 10.3	30.7 ± 10.3
WC (cm)	101.2 ± 10.0	101.7 ± 8.4	107.1 ± 12.5	106.2 ± 11.4
SBP (mmHg)	129.0 ± 9.5	123.3 ± 8.9	126.7 ± 15.7	119.7 ± 13.3
DBP (mmHg)	78.3 ± 5.4	78.7 ± 7.9	82.7 ± 10.2	76.3 ± 7.4
Total cholesterol (mg/dL)	202 ± 42	202 ± 27	183 ± 70	195 ± 54
Triglycerides (mg/dL)	265 ± 70	380 ± 88	237 ± 220	228 ± 176
HDL-C (mg/dL)	39 ± 11	35 ± 7	35 ± 7	39 ± 7
LDL-C (mg/dL)	109 ± 35	89 ± 46	97 ± 42	109 ± 31
Creatinine (mg/dL)	0.7 ± 0.1	0.7 ± 0.1	0.6 ± 0.1	0.7 ± 0.2
Uric acid (mg/dL)	6.1 ± 1.6	5.6 ± 1.4	5.2 ± 1.6	5.1 ± 1.3

BMI: body mass index; WC: waist circumference; SBP: systolic blood pressure; DBP: diastolic blood pressure; HDL-C: high-density lipoprotein cholesterol; LDL-C: low-density lipoprotein cholesterol.

**Figure 2:**
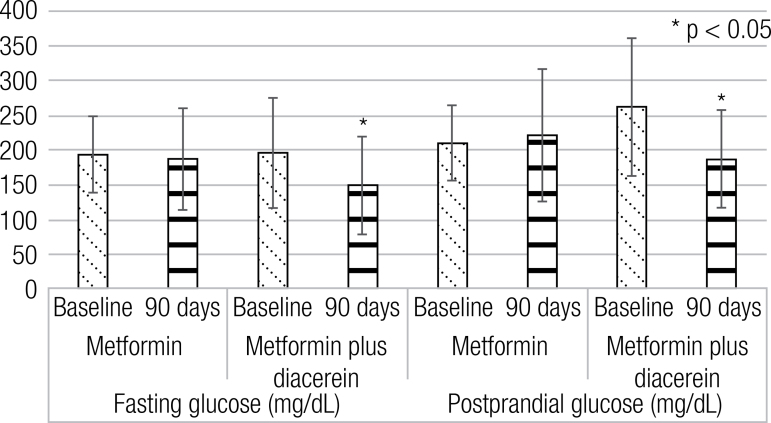
Fasting and postprandial glucose differences before and after the interventions.

**Figure 3:**
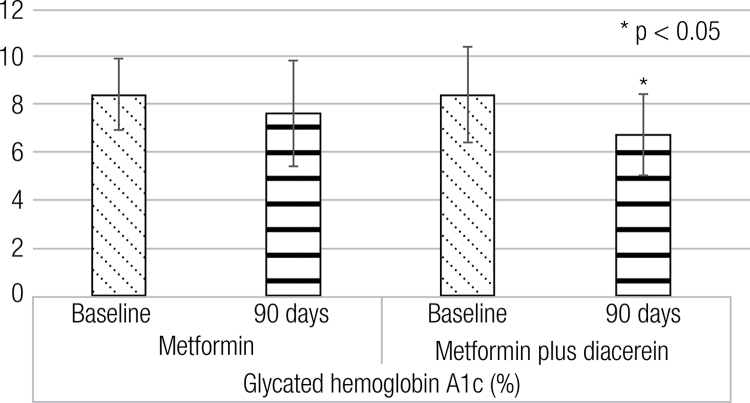
Glycated hemoglobin A1c differences before and after the interventions.

After administration of diacerein as an add-on to metformin, there were significant differences in FG, PPG and A1C concentrations (Table 1, Figures 2 and 3). There were no significant differences after placebo administration.

No serious adverse events occurred during the study. Diarrhea (3 vs. 1 patient, metformin plus diacerein and metformin groups, respectively; p = 0.545) was the unique adverse event reported after the pharmacological intervention.

## DISCUSSION

A low-grade chronic inflammatory state is present in obesity and T2DM (3). Therefore, treating inflammation together with the different described pathophysiological mechanisms of T2DM could be a key point for controlling the diabetes epidemic. The presence of certain pro-inflammatory cytokines such as TNF-α and some interleukins are involved in apoptosis of β cells, which results in a decrease in insulin secretion (14). Use of some pharmacological interventions such as etanercept (15) and some nonsteroidal anti-inflammatory drugs (16) has shown an improvement in β-cell function and insulin secretion by reducing inflammation. In both *in vitro* and murine studies, diacerein has demonstrated to downregulate pro-inflammatory cytokine expression, which improves insulin secretion (7).

These findings led our investigational team to carry out a randomized, double-blind, placebo-controlled clinical trial in 40 drug-näive adult patients with T2DM (10). A hyperglycemic-hyperinsulinemic clamp was used to measure insulin secretion and insulin sensitivity before and after the administration of 50 mg of diacerein for 60 days. The results showed that diacerein administration significantly increases the first, late, and total insulin secretion phases with improvement of FG and A1C levels.

In the present study, diacerein as an add-on to metformin improves glycemic control, significantly decreasing A1C, FG and PPG concentrations. No significant differences were found with the administration of metformin plus placebo.

This may be explained by the findings of a previous experimental study in an animal model of obesity and T2DM treated with diacerein (8). The authors found an important effect on adipose tissue, lowering macrophage infiltration, reducing cytokine production and improving some inflammatory pathways in the muscle and liver. These effects lead to improvement in insulin signaling in the liver accompanied by a reduction in hepatic glucose output and in reductions of glucose concentrations. These effects may be potentiated by the co-administration of metformin that acts according to similar pathways. No significant differences were found in other metabolic parameters such as lipid profile, which coincides with our previous findings.

The main limitation of our study is the small sample size. Further studies with additional patients are needed to confirm our findings. Also, it would be appropriate to more rigorously control certain confounders such as age. In the present study, patients in the metformin plus diacerein group were, on average, 10 years younger than in the metformin plus placebo group.

This clinical trial should be considered as a pilot study for future studies examining the effect of diacerein in combination with other antidiabetic agents.

In conclusion, diacerein as an add-on to metformin in patients with T2DM showed an improvement in glycemic control.
